# 50 Resuscitation with Plasma Does Not Worsen Hypercoagulability or Hyperfibrinolysis in Burn Patients

**DOI:** 10.1093/jbcr/iraf019.050

**Published:** 2025-04-01

**Authors:** Amanda Soo Ping Chow, Anthony Pusateri, Tuan Le, Matthew Gissel, Thomas Orfeo, Maria Cristina Bravo, Melissa McLawhorn, Lauren Moffatt, Jeffrey Shupp

**Affiliations:** The Burn Center at MedStar Washington Hospital Center; Naval Medical Research Unit San Antonio; MedStar Health Research Institute; University of Vermont; University of Vermont; University of Vermont; MedStar Health Research Institute; MedStar Health Research Institute; MedStar Washington Hospital Center

## Abstract

**Introduction:**

Burn-induced coagulopathy (BIC) is characterized by activation of both coagulation and fibrinolysis. A procoagulant shift can be characterized by increased thrombin-antithrombin (TAT) complex levels, reflecting increased thrombin generation. Fibrinolytic function can be evaluated by the ratio of activators and inhibitors of fibrinolysis as evidenced by increased release of tissue plasminogen activator (tPA) and its inhibitor, plasminogen activation inhibitor type 1 (PAI-1). Burn patients who receive more crystalloid fluids than estimated may experience edema-related morbidities. Colloids, such as fresh frozen plasma (FFP), have been used as effective volume expanders to maintain hemodynamic stability during burn shock resuscitation. However, the effect of administration of FFP on the coagulation and fibrinolytic systems of burn patients is unknown. We investigated the procoagulant and fibrinolytic changes in burn patients who undergo resuscitation with FFP, as evidenced by the above plasma biomarkers and their ratios.

**Methods:**

Patients with ≥20% TBSA burns resuscitated with FFP (n=29) were included in this prospective study. Demographics and injury characteristics were recorded. Blood samples were collected at four times throughout the initial burn resuscitation (baseline - within 4 hours of admission; pre-plasma - immediately prior to administration of the first unit of FFP, post-unit - immediately after administration of the first unit of FFP; and post-plasma - after all consecutive FFP units were administered). Concentrations of TAT, tPA, active PAI-1 and the inactive tPA-PAI-1 complex were quantified using ELISA and compared among the four time points using Friedman’s test.

**Results:**

Twenty-two patients (75.9%) were male with a median (IQR) age of 46 years (36-58) and sustained TBSA burn of 34.0% (27.3-48.5). The overall mortality was 27.6%. Across all time points, TAT, tPA, active PAI-1 and the inactive tPA/PAI-1 complex were elevated compared to normal reference ranges. TAT levels did not differ pre-plasma to post-unit but were lower post-plasma [4.8 (4.0-6.9) ng/mL], than baseline [11.5 (8.8-16.6) ng/mL; p< 0.0001], or pre-plasma [8.8 (6.3-13.5) ng/mL; p=0.03]. Compared to baseline [491.1 (329.8-1232) pM], active PAI-1 levels were higher at pre-plasma [1963 (310.9-3866) pM; p=0.04], post-unit [1441 (525.5-4884) pM; p=0.01] and post-plasma [1914 (1498-3536) pM; p=0.02] but did not change between pre-plasma and post-unit. Levels of tPA, inactive tPA/PAI-1 complex and the ratio of active PAI-1 to tPA were unchanged at the four time points (p>0.05).

**Conclusions:**

There was no evidence of worsening sub-clinical markers associated with a coagulopathic or fibrinolytic state after plasma resuscitation.

**Applicability of Research to Practice:**

FFP can be used as an adjunct during burn resuscitation without worsening coagulo-fibrinolytic status following severe burn injury.

**Funding for the Study:**

US Department of the Army W81XWH-19-2-0061

